# Sign and Human Action Detection Using Deep Learning

**DOI:** 10.3390/jimaging8070192

**Published:** 2022-07-11

**Authors:** Shivanarayna Dhulipala, Festus Fatai Adedoyin, Alessandro Bruno

**Affiliations:** 1 Department of Computing and Informatics, Bournemouth University, Talbot Campus Poole, Poole BH12 5BB, UK; s5229871@bournemouth.ac.uk; 2 Department of Biomedical Sciences, Humanitas University, Via Rita Levi Montalcini 4, Pieve Emanuele, 20072 Milan, Italy

**Keywords:** CNN, LSTM, confusion matrix, british sign language, precision, recall

## Abstract

Human beings usually rely on communication to express their feeling and ideas and to solve disputes among themselves. A major component required for effective communication is language. Language can occur in different forms, including written symbols, gestures, and vocalizations. It is usually essential for all of the communicating parties to be fully conversant with a common language. However, to date this has not been the case between speech-impaired people who use sign language and people who use spoken languages. A number of different studies have pointed out a significant gaps between these two groups which can limit the ease of communication. Therefore, this study aims to develop an efficient deep learning model that can be used to predict British sign language in an attempt to narrow this communication gap between speech-impaired and non-speech-impaired people in the community. Two models were developed in this research, CNN and LSTM, and their performance was evaluated using a multi-class confusion matrix. The CNN model emerged with the highest performance, attaining training and testing accuracies of 98.8% and 97.4%, respectively. In addition, the model achieved average weighted precession and recall of 97% and 96%, respectively. On the other hand, the LSTM model’s performance was quite poor, with the maximum training and testing performance accuracies achieved being 49.4% and 48.7%, respectively. Our research concluded that the CNN model was the best for recognizing and determining British sign language.

## 1. Introduction

### 1.1. Background

Human beings have long found it necessary to solve issues that threaten their survival or well-being. As a result, there has always been a need for them to communicate. A significant component required for successful communication is language. Language has been used for a very long time in the expression of ideas, feelings, and emotions [1]. This can be accomplished using written symbols, gestures, or vocalizations. Although the use of language for communication has helped solve problems, it often faces challenges as well. For instance, effective communication generally requires that all involved parties understand and respond to at least one common language [2]. This is not always the case; in specific instances, communicating parties may rely on different written symbolic, sign, or vocal languages. Alternatively, people may have limitations in terms of not being able to read or understand written symbols or vocals, and can be taught to communicate using these methods.

In other cases, human beings may be born with or develop a disability that may limit them from sharing certain forms of language. For instance, people with hearing impairment and people who cannot physically speak due solely to certain disabilities may be limited to the use of gestures and sign language. However, it should be noted that the use of specific gestures or sign languages is not universal, and varies from one region to another and among different ethnic communities worldwide [3]. In addition, learning multiple sign languages is complex and may not be possible for a majority of the public [4]. It is impossible for people with speech impairment to learn spoken language; this means that it is a problem for hearing-impaired people both to communicate with other people who are not conversant with sign language and to communicate among themselves.

Human beings have adopted various methods to solve this challenge. For instance, human sign language translators are commonly used in public places and TV channels to communicate spoken messages in a form that people living with these disabilities can understand [5]. However, in certain cases human sign language translators may not be available, or may not be efficient and reliable. In such cases there is a need to adopt more reliable means of translating sign language into a written or spoken language.

The rapid increase in the use of computers and artificial intelligence [6,7,8] has made it easier to solve cognitive problems [9], including those involved in sign languages and their limitations [10]. This is mainly thanks to the use of digital recognition models that can detect and convert different sign languages and convert them to a form that the public can understand [3]. The application of deep learning to train computer systems to recognize, interpret and, translate signs into written language is one of the most significant trending issues in artificial intelligence [11]. Therefore, this dissertation will consider the application of LSTM and CNN models in sign language and human action detection using deep learning to close the language gap between the deaf and the public. Much progress has been witnessed in Human Object Interaction, which is used to recognise the verbs in which the model is trained on entities of humans and Objects for each, such as <Human, eat, apple>. A cascaded model, a collection of Relation-Ranking and Relation-Classification models, has been developed to collect human semantics and facial patterns and to rank them using a Relation-Ranking Model (RRM). Top ranks from the RRM model are fed to the RCM model to predict the verbs, which can be the trained labels [12]. Another approach to tackling human semantics, an instance human-aware model, has been developed to parse the human semantics and jointly estimate human poses [13].

### 1.2. Problem Statement

Scientists, researchers, and scholars are responsible for propelling humanity by solving problems, eliminating barriers to problem-solving, and promoting cohesion and development in society. A significant problem or barrier to problem-solving lies in ineffective communication and high communication barriers. This problem exists between speech impaired people, who can only use sign language, and other community members who cannot understand sign language. The same issue exists with respect to hearing-impaired people from different regions in the world which use foreign sign languages. This communication barrier problem commonly occurs in public institutions when speech-impaired people seek services from a public service worker unfamiliar with a particular sign language. It limits speech impaired people from working in different places to communicating with sign language interpreters who are not conversant. This limits speech-impaired people from accessing or offering public services and from working in various industrial sectors. While efforts have been made to solve this challenge, for example, the use of human translators, they are not very efficient. The advancement of technology has made it possible to use more advanced systems such as machine learning. This includes the use of special algorithms that facilitate sign language translation into written text that is universally understandable. However, research remains in progress, and it has not been established which model can best solve the problem.

Therefore, there is a need to develop and compare the performance of different machine learning models in the recognition and translation of sign language and human actions for effective communication. This is important because it can enhance the effectiveness of communication between speech-impaired people, regular community members, and those who use different sign languages. It can enable speech-impaired people to enjoy equal opportunities for work in public institutions and various industrial sectors with other people without this disability.

### 1.3. Motivation of the Study

Effective communication is a significant component that we as human beings require to avoid and solve problems. In most cases, inability to communicate properly is due to lack of common language between the communicating parties, which is an essential tool in effective communication [2]. This problem has existed for many years, especially with respect to the need for people from the active speech-impaired (dumb and deaf) community to communicate with other members of society. Although people can use gestures to solve this problem, it is often not practical and a lot of time is wasted conveying a simple message [14]. This happens in many cases and in many different fields, inconveniencing people from the active speech-impaired community. For instance, people may be unable to communicate messages to the authorities [15]. In other cases, they may be hindered from participating in various economic activities such as agriculture despite having all of the necessary skills and abilities due to their inability to communicate effectively with others [15].

A shared sign language (as opposed to communicating through gestures) could be the best way to solve this problem, as it is one of the most common tools used to this end. However, over 120 sign languages are used worldwide, and it is not straightforward for society at large to learn these languages and communicate effectively using them [4]. Researchers and engineers have tried to develop sign language recognition systems to narrow this gap, for example, a conduct-based system known as a glove sensor [16]; however, these systems require hardware setups that are complex and relatively expensive, and therefore they are not preferred [15]. Researchers and engineers have started applying vision-based systems, which are relatively cheaper as they only use cameras [17]. Although many researchers have been venturing into this field, a complete solution to this problem has not yet been achieved [16]. Therefore, this research seeks to develop an efficient deep learning model able to detect and understand British sign language as well as to determine which learning model among different alternatives can best perform sign language prediction.

### 1.4. Scope of the Study

The main goal of this research is to develop an efficient deep learning model that can be used to detect, understand, and translate British sign language to written text. Two models were developed, an LSTM [18] model and a CNN model [19]. Their performance was evaluated and the results compared to determine the best model. Two approaches were applied in developing the models, differing based on the type of data used. The first approach involved importing pre-processed data from Kaggle, a platform where experiment datasets are freely available to the public [20]. The second approach involved collecting data from a computer webcam using a computer vision algorithm and extracting key points, including hands, face, and pose. As a sequence, these key points can then be passed along to detect and decode actions and sign language. To achieve this, the model’s artifact provides an “h5” weight file for a model that is then applied in deploying and testing the model using images of different sign languages and producing a text output on a webcam. To evaluate the objects of this research, we applied multi-labelled classification. This supervised learning prototype involves assigning each data instance several labels from a predefined set of tags. This trending approach is used where the available dataset is too complex for each instance to have a specific class.

## 2. Literature Review

This section contains information obtained from the literature about previous research related to applying deep learning models in the context of sign language and human action recognition. The chapter is divided into section dealing with an overview of sign language and human recognition, the need for sign language and human action recognition, related works, research gaps, and the conceptual framework. The related works section contains existing information about the available alternate models used for sign language and human action recognition as well as a critical analysis of their applications and limitations and recommendations as to possible future studies.

### 2.1. Overview of Sign Language and Human Recognition

Sign language recognition, referred to as SLR, can be defined as the detection and systematic conversion of various sign language gestures to a language that the general public can understand, such as words, symbols, or speech [15]. These sign language gestures consist of hand movements, facial expressions, and even the movement of the entire body. Movements are structured in different forms with different meanings to achieve all of the same goals achieved by standard active spoken language. These goals are the expression of personal feelings, desires, opinions, and ideas [21]. On the other hand, human action recognition involves applying data sensors in order to predict a person’s movements as well as processing certain important inferences [22]. Integrating these actions and different sign language gestures with human–computer interface models contributes to the research around human action recognition. The human–computer interface approaches used in sign language recognition generally consist of either conduct-based or vision-based systems. In the former, the person performing the sign language gesture is usually physically using a human–computer interface device that captures their actions and sends them to the other hardware setups. Using a special algorithm housed in the setup, the received signals are then converted into the desired output. An excellent example of conduct-based sign language recognition is the sensor glove [17]. In vision-based systems, on the other hand, the person performing the sign language recognition is not in physical contact with the human–computer interface data capture device. Instead, their actions are captured using a pre-programmed camera, for instance, a computer’s webcam. These data are then processed using a special algorithm to obtain the desired output form and the results are projected by the respective output device. A good example of the above approach is the application of deep learning, which makes human action recognition more suitable and appealing [17]. It should be noted that while sign language recognition is a relatively complex subdiscipline of data science, there are numerous studies have been successful in achieving more efficient sign language recognition prototypes.

### 2.2. Need for Human Action and Sign Language Recognition

Human actions and sign language recognition are among the trending issues in data science and artificial intelligence, and have many applications [23]. The initial and most prioritized goal [15] for the development of human action recognition is to narrow the communication gap between the active speech-impaired community and the general public [24]. Action recognition is to narrow the communication gap between the active speech impaired community and the regular public members [25]. This will enable different community members to live in harmony thanks to the greater ease of sharing feelings and personal expression. The narrowing of this gap will be very useful in helping more people to better share their ideas and knowledge, aiding different production sectors such as industry and agriculture [15]. The application of sign language and human action recognition has found many applications in different areas over time. For instance, the technology can now be applied in public places such as airports, churches, and hotels. Educational institutions can use this technology to facilitate learning activities and interactions between hearing-impaired and visually impaired students [26]. In addition, sign language and human action recognition have been applied in computer and mobile phones, gaming interfaces, machine control, robotics, and televisions [27]. Sign language and human action recognition can be applied for easy interaction between children and computers, development of recognizable forensic identification, and video conferencing communication [28]. This shows the need for the continuation of research to advance human action and sign language recognition.

### 2.3. Related Works

Several scholars, scientists, and engineers have conducted various kinds of research regarding human action and sign language recognition. Although few have examined conduct-based models, the vision-based approach has been considered by many researchers due to its efficiency and cost. In this section, several pieces of research related to sign language and human action recognition are reviewed and a critical analysis conducted of the proposed or tested models in the aforementioned studies. This will help to develop and explain the specific research gap on which the present research is focused. In the effort to narrow the communication gap between the deaf community and the hearing community, different solutions such as hearing aids have long been the primary consideration [21]. Over time, two main approaches have been applied to the development of sign language and human action recognition models, namely, the conduct-based and the vision-based model [16]. In the conduct-based model, human actions and sign language gestures are captured using hardware devices or sensors that are in contact with the person communicating. The capture signals are sent to a computer or complex of computer hardware setups and then decoded using a special predefined algorithm. An excellent example of these models is the glove-based device developed over three decades ago [16]. This device has a number of drawbacks. For instance, the translation accuracy is very low, as the sensor gives incorrect predictions over time. The wearable devices were not personalized for specific users; rather, they detected gestures and converted to text for specific predefined settings. They did not have a mechanism for storing sensor values for future use in extended data analysis [16]. Using similar principles and applying arterial neural networks, an improved conduct-based sign language prediction model was developed that translated the captured gestures to speech [16]. This model used flex sensors attached to a special glove that detected the wearer’s gestures. The flex sensors were set to adjust several resistance values using the angles produced by specific flex actions, altering the voltage. Through several mechanisms, different values were displayed [16]. This improved model was found to be susceptible to various errors. The model seems too complex to develop, as different flex angle for the same action may vary from person to person and even for one person due to various factors. It has been noted by different scholars, scientists, and engineers that conduct-based models usually apply relatively expensive and complex hardware setups. According to many scholars, vision-based models seem to be a more reliable, relatively inexpensive, and efficient approach that best fits sign language recognition [17]. In this approach, sign language gestures are captured using a camera mounted on a computer, such as a webcam, then decoded using machine learning principles. Different scholars, scientists, and engineers have explored several vision-based models to establish the most reliable and efficient model. A vision-based real-time sign language recognition system was proposed for translating southern Indian sign language. This device has a couple of drawbacks. For instance, the translation accuracy is very low as the sensor gives wrong predictions with time. The wearable devices were not personalized for specific users but rather detected gestures and converted to text for specific predefined settings. The dives also did not have a mechanism for storing sensor values that could be used for extended data analysis [24]. Using similar principles and applying arterial neural networks, an improved conduct-based sign language prediction that translated the captured gestures to speech [24]. This model used some flex sensors attached to a special glove that was to be worn by the person communicating to detect the gestures. The flex sensors were set to adjust some resistance values by the angles produced for specific flex actions, altering the voltage.Through some mechanisms, different values were displayed [24]. This improved model was also found to be susceptible to various errors. The model also seems too complex to develop as the different flex angle for the same action may vary from person to person and for one person due to some factors. It has also been noted by different scholars, scientists, and engineers that the conduct-based models usually apply relatively expensive and too complex hardware setups. According to many scholars, vision-based models seem to be a more reliable, relatively inexpensive, and efficient approach that best fits sign language recognition [17,29]. In this approach, sign language gestures are captured using a camera mounted on a computer, such as a webcam, then decoded using machine learning principles. Different scholars, scientists, and engineers have explored several vision-based models to establish the most reliable and efficient model. A vision-based real-time sign language recognition system was proposed for translating southern Indian sign language [30], while a finger spelling method was developed in [19]. The authors in [30] applied 32 signs representing finger positions defined Using “UP” and “DOWN” binary representation. Using fingertip positions, the sign language captured through the images was converted into text. A single user was used in both the development and testing of the model. The proposed model attained a 98.125 accuracy with 50% of the 320 images used in training being tested. The authors suggested that more accurate measurement of angular movements of different gestures could be applied to improve the accuracy of their system. Another hand gesture sign language recognition study used a hidden Markov model in context-sensitive research [31]. The Hidden Markov is a machine learning model used for speech recognition and task classification which offers solutions to problems involving evaluation of data, decoding, and learning by determining the most appropriate classifications. From their research, the authors concluded that the hidden Markov model had better performance than the previous models due to their higher statistical accuracy and the ease of performing further modification. The authors’ hidden Markov model easily accommodated new posture classes and removed existing classes while retaining the required ones. The model was able to apply Self-Organizing Feature Maps (SOFM) and a Single Recurrent Network (SNR). The SOFM was used as feature extraction for continuous HM to transform input signals into low-dimensional representation for easy modelling. The SNR was then used for segmentation of constant SLR to inform the transformation of the presented SOFM. Their model attained an 82.9%-word recognition rate and an 86.63% continuous signer-independent sign language recognition rate. The authors recommended further research on the effective extraction of features from different signers, general model compact training sentences, extraction and minimum definition of units in sign language recognition, use of statistical sign language models, and utilization of automatic sign language parameters. Another vision-based sign language recognition system was developed for automatic translation of Arabic sign language from signs to text. The system consisted of four main stages: segmentation of the hand by use of a dynamic skin detector, tracking, extraction of features, and classification of data. The results obtained from the experiment showed a 97% signer-independent recognition rate and outperformed the pre-existing models by the accurate specification of the hand and heads positions, respectively. Deep learning CNN has nowadays gained much popularity in data science research, including the application of vision-based hand gesture recognition for sign language interpretation using deep learning [27]. In one study, a Deep learning-based CNN that was specifically designed for sign language recognition was used. VGG-11 and VGG-16 were trained and tested in the research for the evaluation of model performance. The authors used a sizeable Indian sign language collection with 2150 images obtained using an RGB camera and an ASL data set. The Indian sign language model obtained 99.96% accuracy, while the ASL model obtained 100% accuracy. Other efficiency tests apart from accuracy were conducted on the model to compare the models with the most advanced approaches. The findings from the tests showed that the study model performed better than the existing models and showed more significant potential for improvement than the rest. However, they= authors acknowledged the existence of several errors and the possibility of failure with different regional sign languages. Therefore, they proposed that future research focus on optimizing hand gesture recognition as well as on additional comparisons and improvements to the architecture intended to minimize errors.

Sign language recognition research was conducted by [32] to eliminate the communication barrier between the deaf community and the hearing community. Microsoft Kinect CNN models using GPU acceleration were applied in this research. Feature construction was automated using CNN models [32]. Using the model, 20 different Italian sign language gestures were recognized with high accuracy. Cross-validation accuracy for the predictive model was 91.7% based on general user surrounding that did not occur during the training. The model achieved a score of 0.789 on the Jaccard index during the Chalearn spotting competition in 2014, which involved the detection of people’s gestures. The study concluded that CNN could perform accurately when using indifferent sign language recognition that has some users and surroundings uninvolved in training. The authors therefore recommended the use of CNN models for automatic sign language recognition. Similarly, a CNN was used in the development of Bhutanese sign language digits [4]. This model used about 20,000 sign images for Bhutanese sign language recognition of ten static digits obtained voluntarily from a different participant. The research involved a comparison of other sign languages with the proposed CNN model. From the comparison, their proposed model attained a training accuracy of 97.62% for testing and 99.94 for training accuracy. The authors evaluated the model’s precision, Fl-score, and recall and concluded that the misclassification and testing accuracies depended on the number of images in a data set. It was possible to further modify the accuracy using transfer learnings such as ResNet, MobileNet, and VGG16. The authors’ recommendation for future research was that the dynamic gestures and alphabets of the Bhutanese sign language be studied further. Likewise, Ref. [27] developed a sign language recognition model using CNN to translate Indian sign Language to text. This research was termed the first comprehensive analysis of Indian sign language. The study proposed using a three-layer CNN Model which was trained from an absolute initial machine learning state and attained a recognition accuracy of 99.8% on the Indian sign language numerals and 97.8% on the alphabet. The recommendations from the research were that further studies focus on using comparative analysis for the selection of suitable sign language recognition models. Finally, an Arabic sign language classification system was proposed by [25]; their proposed model consisted of a Convolutional Neural Network integrated with an attention mechanism for retrieving spatial features and bio-inspired deep learning with Long Short-Term Memory (BI-LSTM). The BI-LSTM was used for temporal feature extraction. The testing of this model involved using highly variable characteristics such as variable lighting conditions, different clothing, and different distances from the camera. The model that emerged consumed less processing time than the alternatives thanks to the processing of fewer deep learning layers and fewer parameters. Future research recommendations from the authors included testing other domains such as EEG and image classification using their proposed models.The low thermal image dataset, collected from multiple authors with 32 × 32 resolution, has been updated to correspond to 0–9 sign language digits using a high thermal image captured using a light-independent thermal camera that produces an array of 19,200 pixels with 160 × 20 resolution [33]. The employment of CNNs and thermal infrared images for hand 340 gesture recognition is tackled in [34].

### 2.4. Research Gap

One of the main points gathered from the above research is that conduct-based sign language recognition approaches are not preferable. This is because they require relatively complex hardware setups which are rather expensive [17]. This explains why the majority of researchers have been inclined towards vision-based models. Although many studies have been conducted in this field, it is evident that the development of sign language recognition models remains a complex field of research. The most challenging element is developing a suitable model for solving continuous sign problems that are signer-independent [35,36]. There is considerable variation in duration, speed, and background from one signer to another, which poses a challenge to the development of a model with high accuracy and that is continuous from one model to another [25]. Different researchers have provided various recommendations for further research into CNN-related models. However, another challenging aspect lies in determining the best model to adopt and improve, as the interdependence of different models with respect to the optimization of tuning parameters must be considered [27]. From the above literature review, it is evident that most of the CNN models to date have been developed for Indian, Arabic, and Chinese sign languages, among others. However, there has not been much research on British sign language recognition development using the CNN and LSTM models. There are no studies comparing the performance of LSTM and CNN models for British Sign language recognition. This shows a need for study on ways to increase the accuracy of future models by controlling the affecting parameters as well as on developing a reliable evaluation method for determining the most effective model. Therefore, this research focuses on developing a system for British sign language recognition that applies CNN and LSTM while optimizing accuracy via control of the affecting parameters. The study then compares the performance of the two models using the appropriate mechanism.

### 2.5. Conceptual Framework

This section provides an overview of the approaches applied in this research to solve the problem of sign language recognition. In addition, the variables considered in this research are identified and their expected relationships are established.

#### 2.5.1. Overview of The Approaches

**Figure 1:**
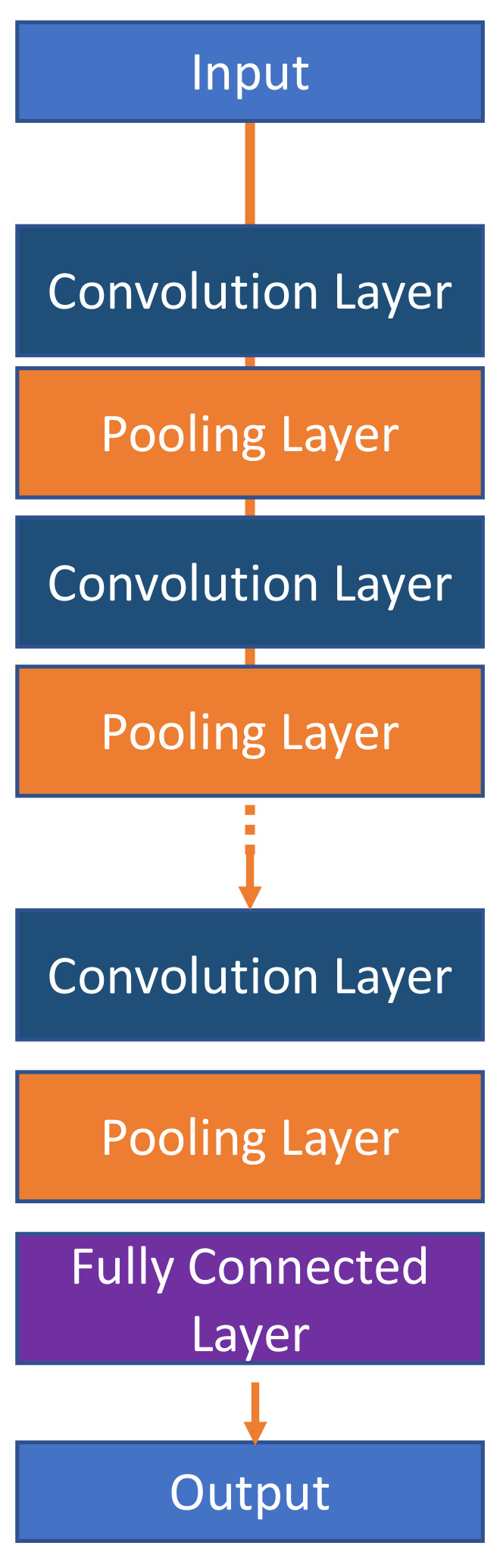
CNN conceptual model (Retrieved from [40]).

The two main approaches considered in this research include the use of the CNN and LSTM models [37,38]. The CNN model is a significant part of the Neural network used to recognize and classify images during the detection and recognition of signs and faces [9]. CNN models are made up of neurons that have learnable weights as well as biases. Specific neurons receive input data and weighted sums are taken over, activating certain functions and generating certain outputs in response to actions. CNN models are commonly applied in multi-channelled images. These models mix the red, blue, and green colours of an image and generate a simple colour spectrum perceivable by the human eye. The CNN has three main layers to accomplish this operation, namely, the convolutional, action, and pooling layers. The convolutional layer applies kernels to capture the characteristic product of filters in the images and sums the values for every action slide. It usually detects useful features such as corners, edges, and intensity lines [39]. The action layer then applies a Rectified Linear Unit to enhance the non-linearity from the initial step. The pooling layer is then applied to the down sample feature and disapplied in all the 3D volumes. A conceptual framework showing the most representative layers of Convolutional Neural Networks is presented in Figure 1.

Unlike a CNN, an LSTM is a recurrent neural network used to process and predict particular data sequences. The main difference between an LSTM and a CNN is that the latter is applied to evaluate the spatial correlation of the data. The LSTM consists of neurons that feed themselves without a predeceasing stage being in place as the input for the following procedure in the same sequence [39]. Another distinguishable feature of an LSTM model is that it possesses a forget gate that enables it to accommodate new user data and forget unwanted data while retaining any required data.

#### 2.5.2. Variables

**Figure 2:**
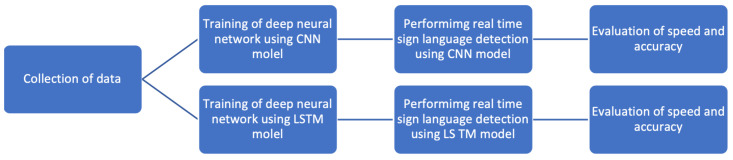
Conceptual Framework.

The leading independent variables in this research were the LSTM models and CNN models used for sign language and human action recognition. The different models were trained and tested to recognize different sign language and human action gestures, and the results were compared between the two. The dependent variables under comparison were the speed and accuracy of the different sign language and human detection models. The relationship between the variables is shown below in Figure 2.

## 3. Research Methods and Planning

This section provides a more detailed discussion and analysis of the CNN and LSTM models’ respective architectures, experiment designs, and required data. Further discussion of the method of implementation and evaluation of the two models is provided as well for comparative purposes.

### 3.1. Methodology

The proposed research experiment was aimed at developing a more efficient deep learning model for accurate detection, decoding, and translation of British sign language into a text message format. The proposed model uses a specially coded computer vision algorithm to detect sign language gestures from a suitable source. The collected data Key points are then processed in sequence for action detection and decoding of British sign language. Two models were be developed, namely, a CNN and an LSTM sign recognition model. The two models were then evaluated using a multi-labelled classification model, a supervised learning prototype in which data are assigned various labels from a predefined set of labels.

### 3.2. Architecture of the Proposed Models

#### 3.2.1. CNN Architecture

**Figure 3:**
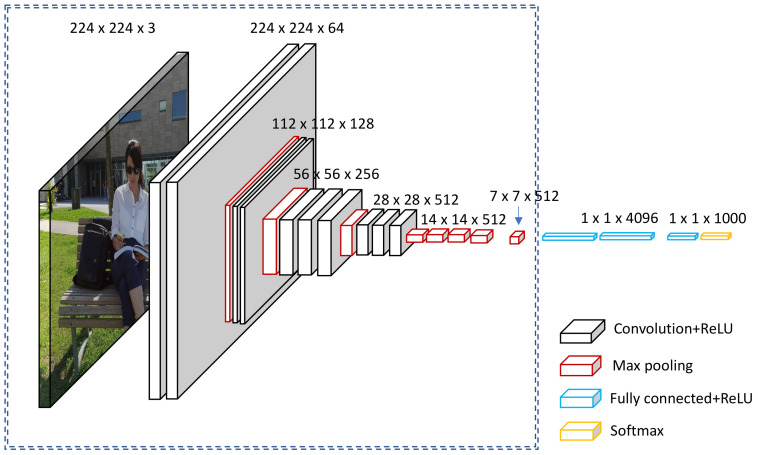
Above a graphical depiction of Convolutional Layer + ReLU and Max Pooling is given (Retrieved from [42]).

A CNN deep learning model is designed to process image inputs specifically. Therefore, they have a specific architecture that is composed of two major blocks. The first block mainly serves as a feature extractor; therefore, it is considered the distinctive feature of the CNN [41]. The extraction of the image features is achieved by matching templates using a convolutional filtering process. The image is filtered by the first CNN layer using several convolution filters, and the return feature is resized or normalized using the activation function. This procedure can be repeated. The obtained feature maps can be filtered with Kernels to produce new feature maps for normalization and resizing, and the process is repeated several times. The values of the final feature maps are sequenced into a vector which defines the output of the first block. This output of the first block is the input of the second. Figure 3 below shows a diagrammatic representation of this, with the first CNN architecture block designated by the circled section on the left side.

**Figure 4:**
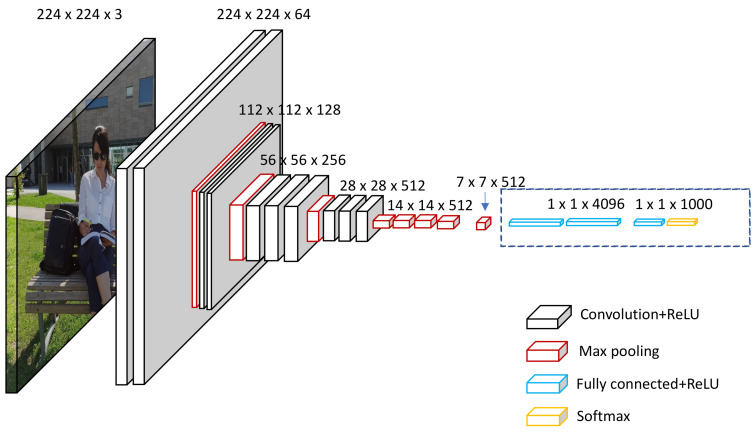
In this picture, the latest four layers of the CNN are shown, respectively, fully connected+ReLU layers and Softmax Activation Function. (Retrieved from [42]).

The second block bock appears at the end of the CNN model, as in all classification neural networks. Using several activation functions and linear combinations, the values of the input vectors are transformed to a new vector as the output. The last vector is made up of multiple elements such as classes. All the elements range from 0–1 (where element “i” is a probable representation that the images lie under class “i”), and their total sum is 1. The probabilities are calculated based on the final layer of this block using a logistic function or SoftMax functions, which are binary and multi-class classifications, respectively. A back-propagation gradient is used to determine the layer’s parameters, and cross-entropy is reduced during the training stage. Figure 4 below shows a diagrammatic representation of the second CNN architecture block, designated by the circled section on the right side.

**Figure 5:**
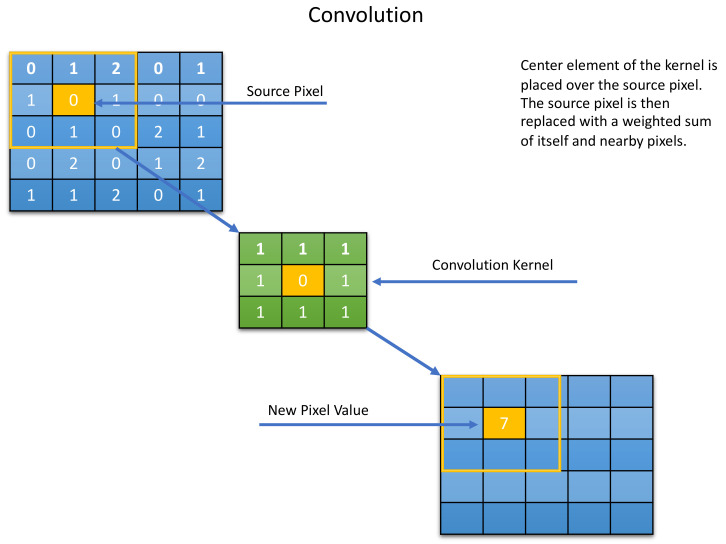
Above, the so-called convolutional layer is broken down into its elementary components.

In addition to the architectural blocks, the CNN has three layers, namely, the convolutional, pooling, and the ReLu (Rectified Linear Units) layers. The purpose of the convolutional layer, which is the first layer, is to detect the sets’ features present in the input images. This is achieved through filtering, which involves dragging a window that represents images’ features and calculating the convolutional products between them. At this stage, the feature are termed as filters, as the two are now seen to be equal. Several images are received by the convolutional layer and their convolutions are calculated using each filter that correspond to the required features in the images. Feature maps (image, filter) are then obtained; these represent the location of the features in a particular image. It should be noted that high coordinate values indicate a high degree of resemblance of image and features. Figure 5 below is a representation of the convolutional layer.

The pooling layer is the second layer in the CNN model; its function is to receive various feature maps of images and reduce their sizes while maintaining valuable characteristics. This is achieved by cutting the images into smaller regular cells to minimize information losses while retaining the maximum values within each cell; 3 × 3 and 2 × 2 cells are the most common type that either overlap or do not overlap. The obtained output is usually the same number as that of the input feature maps except with smaller sizes. The main purpose of pooling is to improve network efficiency and avoid overlearning by reducing the number of parameters and calculations present. Finally, the ReLu correction layer is an activation function that replaces negative values in input feature maps with zeros. It is a nonlinear function obtained by ReLu (x) = max(0,x) and diagrammatically represented as:

CNN models have another layer, known as the fully connected layer; although not a CNN characteristic, this layer produces input vectors using appropriate linear combinations and activation functions. This layer is known for the classification of input images into the network. It calculates probabilities by multiplying elements by specific weights to make sums. An activation function defined as (logistic if N = 2, Soft-Max if N > 2) is then applied. This is similar to the multiplication of the input vector by the weight matrix. The way that the CNN learns, convolutional layer filter via back-propagation gradient, is similar to learning weight values. This concept can be applied in the evaluation of the model.

**Figure 6:**
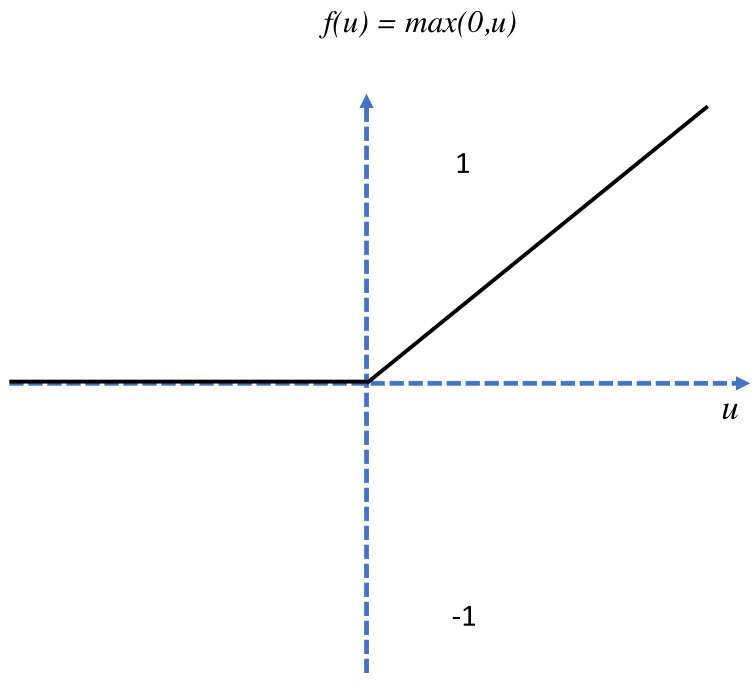
Re-Lu layer.

The curve for the Re-Lu activation function is shown in Figure 6.

#### 3.2.2. LSTM Architecture

Long short-term memory, commonly known as LSTM, is a type of RNN architecture that is capable of remembering values at arbitrary intervals. They are developed for the classification, processing, and prediction of time series of particular time lags with durations that are unknown. Unlike other sequence learning models such as hidden Markov models or other RNNs, LSTMs have relative intensity gaps, which provide an advantage over the alternatives [43].

**Figure 7:**
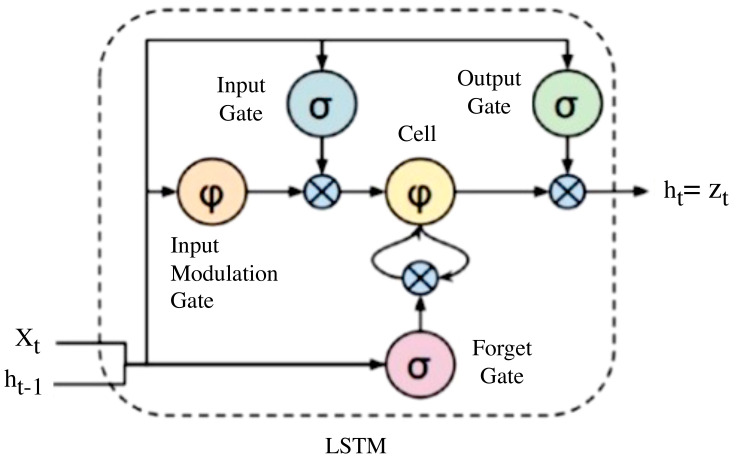
LSTM cell.

A zoom-in on an LSTM cell is depicted in Figure 7.

The LSTM is referred to as the cell state and has a looping arrow that signifies its recursive nature. As a result, the information from the previous interval is stored within the cell state. A remember vector located below the cell state modifies it, while the input modification gates adjust it. This remembers vector is usually referred to as the forget gate.

**Figure 8:**
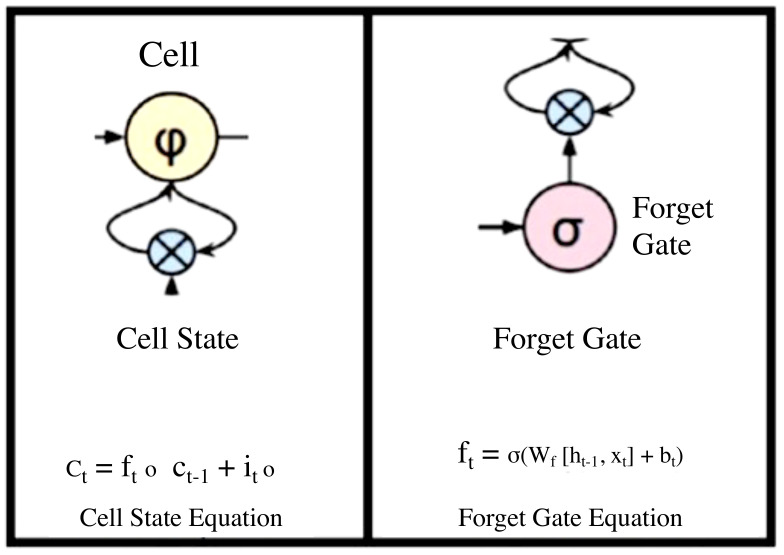
LSTM Cell State and Forget Gate.

A zoom-in on an LSTM cell is depicted in Figure 8.

From the Cell state equation, the information is forgotten through multiplication with the forget gate, and new information is added via the input gates’ output. The information to be forgotten by the cell state is determined by the forget gate through multiplication of a required matrix position by 0. On the other hand, if the value of the output at the forget gate is I, then the information is retained in the cell state. A sigmoid function from the algorithm is then applied to input along with weight and previously hidden state. A save vector, commonly known as an input gate, is usually responsible for the determination of the information that should enter the LSTM. This is a sigmoid function and has a range of (0, 1). This only adds memory and does not forget, as the equation of the cell state is a sum of the previous cell states. The focus vector is referred to as the output gate, while the working memory is known as the hidden state.

**Figure 9:**
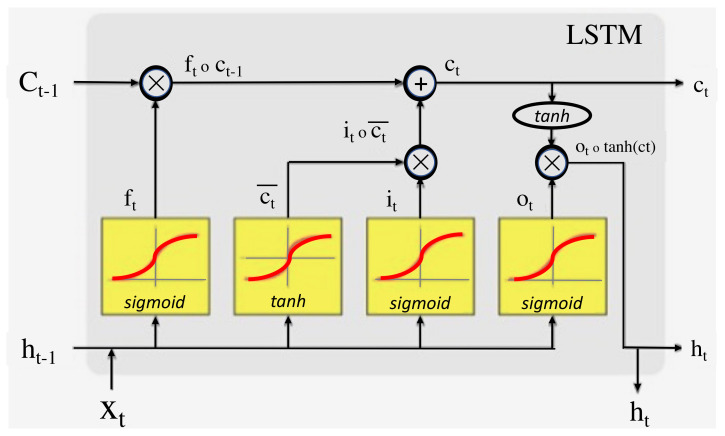
LSTM Cell.

A zoom-in on an LSTM cell is depicted. Sigmoid functions are highlighted here in Figure 9.

In the above diagram, the first sigmoid on the left-hand side is the forget gate and defines the information that the (Ct-1) cell state should forget. The input gate is defined by the tanh activation function and the second sigmoid and determines the information that is to be saved or forgotten in the LSTM. The output gate is defined by the last sigmoid and determines the information that is to proceed to the next hidden state.

### 3.3. Experiment Design

#### Data Description

In this experiment, the required data were about 30 signs used in British sign language and common human action to signify emotions. The signs were based on three categories, namely, hand gesture signs, pose signs, and facial expressions, or on a combination of facial expressions with either hand gestures or pose signs. The experiment applied nineteen British sign language hand gestures for numerals 0–19, nine pose signs for simple common messages, and two facial expressions combined with a pose or hand gesture.

**Figure 10:**
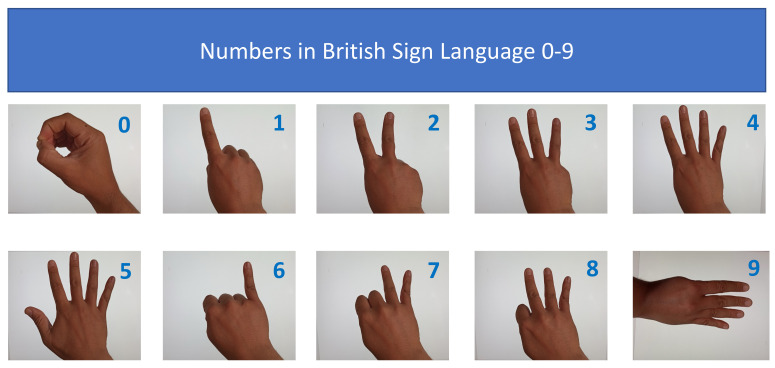
British Sign Language Hand Gestures.

Numeric signals in British sign language are shown in Figure 10.

**Figure 11:**
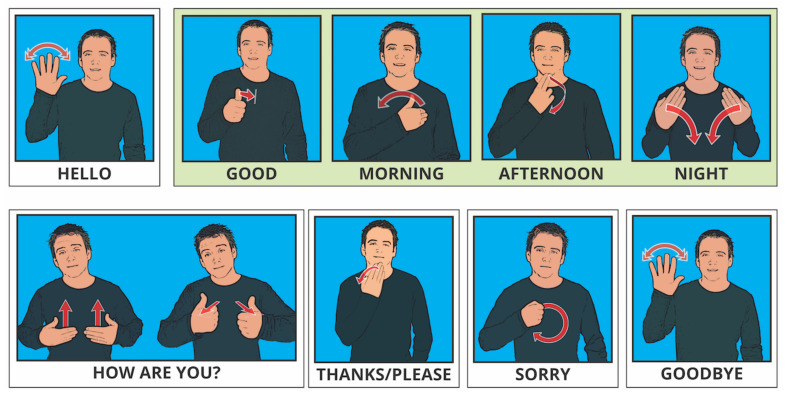
British Sign Language Pose Signs (credits of the picture goes to [44]).

Here are some British Sign Language Pose Signs in Figure 11.

### 3.4. LSTM Model Methodology Design

The main goal of this experiment was to develop an LSTM model to predict British sign language using multiple frames and to predict the action being demonstrated in real-time. The first step was to collect and save data from different key points, that is, the hands, face, and body. The next step was to train the deep learning model using an LSTM layer and using this to predict temporal components, enabling prediction of actions from a certain number of frames. The final step was to use the open CV and predict actions in real time using a computer webcam. To achieve these three main goals, the first requirement was the installation and exportation of dependencies in the python code, followed by the extraction of key points using MediaPipe holistic. This was followed by determining how the extraction of key point values (for instance, joints with the hands or body) would be carried out to represent a different frame at a point in time for the LSTM model. The Keypoint values were then collected for testing and training. These data were prepossessed and sequences created through the creation of labels and features.

#### 3.4.1. Building and Training of LSTM Neural Networks

The first step was the importation of key dependencies, namely, the sequential models, LSTM layer, and dense layer. This was followed directly by the creation of a log and setting up of a Tensor Board callback for monitoring neural network training. The models were then compiled and fitted by specifying the preferred optimizer to be used and the loss functions (categorical crossentropy) for the multiclass classification model. The metrics were specified to trace accuracy during the training process. After this, the model was ready to be fit and trained. This was achieved by defining the X-train and Y-train data and specifying the number of epochs and callbacks. The training of the model was then initiated and the raining accuracy was monitored using the Tensor board. The training was able to be run until the specified number of epochs were completed or stopped when the desired training accuracy was achieved. After predetermination of the probability for each predicted action, the predictions made were evaluated and the weights were saved using the ”h5” model.

#### 3.4.2. Justification for the Used Tools

The main reason for using the MediaPipe holistic combined with the LSTM model was because fewer data were required to produce a hyperaccurate model, fewer parameters were required to enhance the speed, and a simpler model was required for easy detection of actions in real time. According to Amin et al. [45], the LSTM model can produce that.

### 3.5. CNN Model Methodology Design

As with the LSTM, the main goal of developing the CNN model was to develop a more accurate and efficient British sign language and human action detection system. This process consisted of three main stages, namely, data input and extraction, implementation and training, and evaluation. Data extraction involved the use of a computer webcam to extract holistic key points from the key points, that is, the face, hands, and body. Facial landmark detection was applied to obtain holistic key points from the face by localizing different parts of the face, including the lips, nose, and eyes. Hand gestures were recognized using two steps, hand detection and classification. For detection, color filters were applied using thresholds derived from face pixels, providing high accuracy and speed. Ensemble learning was then applied for the classification of steps. Face detection and landmark extraction and orientation of landmarks with an approximation of horizontal and vertical axis were the processes applied for pose actions.

#### 3.5.1. Implementation and Training

The CNN model was trained for the recognition of gestures and their classification output was ensembled using a bagging approach. An open source object detection library known as Dlib (trained over a large set of HoG and SVM) was applied for the extraction of facial landmarks. In general, the training involved four independent deep CNNs using tensor flow and ensembled via a transfer learning process. The final prediction confidence was obtained using the bagging ensembled technique.

#### 3.5.2. Justification for the Used Tools

This method applied ensemble learning because it boosts classification performance and reduces the chance of overfitting by the model during training. Ensemble learning mitigates biases and variance, which negatively impact the classification performance of new data sequences [42]. The hand gesture recognition methodology applied in this research has previously produced high accuracy and speed [42]. In addition to this, training the Dlib over a larger set of HoG and SVM makes it lightweight and adds negligible latency during the processing of input frames, which makes it highly compatible with real-time detection.

### 3.6. Evaluation and Comparison

This research project evaluated both the CNN and the LTSM models using the multi-labelled classification confusion matrix paradigm. In this case, the confusion matrix provides comprehensive intuition into the performance of the multi-class confusion matrix problem classifier. In addition to the computation of precision and recall, this paradigm is a magnifier that offers deeper intuitions into internal classification of classifier operations. In addition, using confusion matrix inspection and its derivatives provides strong clues for the analysis of relationships between labels and classes, which represent semantic meanings and concepts that have been assigned to data instances. The results obtained from the two models, including accuracy, precision, and speed for both cases, were compared. From this, the best-performing model was identified, allowing the necessary recommendations to be given.

## 4. Results

This chapter provides information on the results obtained during the development, training, and testing of the deep learning models. The section covers the extensive illustration, presentation, and interpretation of the data on the production, training, and testing of the CNN and LSTM. The results are classified into two main categories based on the type of data used, that is, numerical sign language detection and simple standard messages (facial expressions combined with pose signs).

### 4.1. Deep Learning Models for Numeric Sign Language Detection

This section covers the results for the deep learning models used to detect ten numerical signs, from 0–9. The data under this section were developed taking pre-processed data sets into consideration. This means that the images’ pixel values had already been obtained and coveted into the form required by the model for integration into the experiment. These pre-processed datasets were obtained from Kaggle [46]. Kaggle is a platform where experimental datasets are freely available to the public [20]. The data were collected by importing the NumPy array from Kaggle.com, where the datasets had already been processed [46]. After all the necessary libraries had been imported, the different images resulting from the NumPy array were then mapped to define their shapes. The dimensions of the selected images were (2062, 64, 64), which means that the length and width of an image’s fame were 64 × 64, respectively, in 2062 rows. These data were usable for both the LSTM and CNN models, as the images had the same length and width dimensions. Using a unique function, the data values were retrieved and applied to understand the problem statement. This was because the algorithm for the data depends on multiple class problems or binary classification problems. The balance between the values was found by factoring out the unit values. Equally-divided classes were generated, showing the dataset to be balanced, an essential factor that can significantly affect the results of both the CNN and the LSTM deep learning models. At this point, the dataset contained nine classes ordered from zero to nine, as shown below.

**Figure 12:**
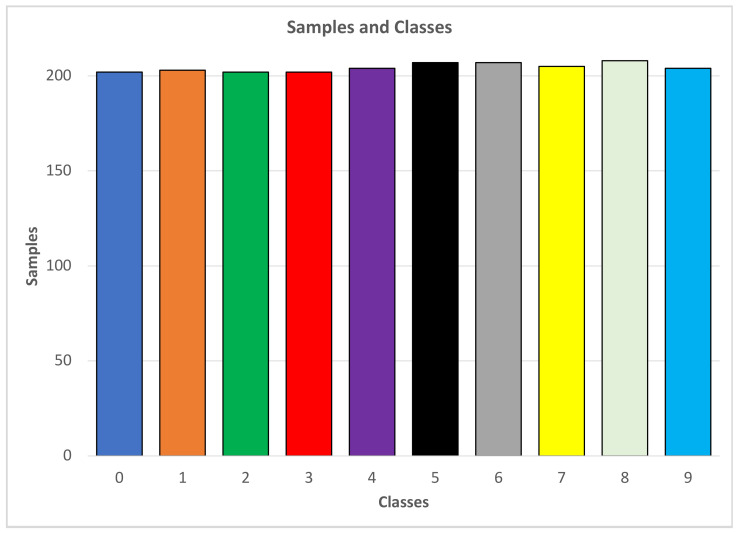
Classes from 0–9.

In Figure 12, all data samples are distributed across ten different classes, corresponding to ten numerical signs in the range [0, 9].

During cross-validation, the dataset was divided into four main divisions. Two divisions were used to train the system, one for testing the design, and the other for validation. The dataset was thus a 70/30 Spector type, in which 70% of the data is used for training and 30% for testing. As convolutional neural networks (CNN) use the full dimensions of the data, that is, the length, width, height, and colour, the data needed to be reshaped. Therefore, the dataset was further processed and reshaped into CNN-compatible data and scaled to convert all the bales into intervals of (0,1).

#### 4.1.1. Building and Training of the CNN Deep Learning Model Using the Pre-Processed Data Set

With the dataset ready for developing, training, and testing the deep learning model, the Convolutional Neural Network was created by fitting the input data above using the appropriate algorithm in python. Using an Adam optimizer and a learning rate of 0.001, the results were compiled and fitted into the CNN model. The training and testing of the model were carried out over the course of 100 iterations.

#### 4.1.2. Evaluation of the CNN Model

Typically, the training accuracy of a deep learning model is expected to be better than the testing accuracy; the difference between the two is not likely to be below 5% in a standard setting. Both the training and the testing accuracy may behave differently throughout the training and testing process. The accuracy may either increase or decrease at different stages of the training or testing process, depending on the nature and performance of the developed model. In this experiment, the training and testing of the CNN model were left to run and recorded for all 100 epochs. The training accuracy was relatively low at the initial point, at 11% in the first and 10% in the fifth epoch. The testing accuracy at this point was around 8.0% for the first five epochs, and the loss was around 2.3. The difference between the training and testing at this point was within the allowable range of 5%. The CNN model showed good performance as the training progressed, with a steep increase in both training and testing accuracy between the 10th and 25th epochs. The improvement then became more gentle, remaining at a recommendable range of above 90% for both training and testing, while the loss continued to decrease. Towards the end of the training and testing, the convolutional neural network was seen to have attained a stable accuracy lying within the range of 97% to 98%. On the other hand, the testing accuracy ranged between 95% and 96% as the training approached the end. The lowest training accuracy in the model was 7.9%, while the highest training accuracy was 98.8%, for a range of 90.9%. On the other hand, the lowest testing accuracy was 8.0%, while the highest testing accuracy was 97.4%, providing a range of 89.4%. The figure below illustrates the behaviour of the testing accuracy in both the training and testing of the convolutional neural network during this experiment. The accuracy is plotted on the *Y*-axis in the graph (ranging from 0–100%, where 1.0 represents 100%). In contrast, the number of epochs is plotted on the *X*-axis from the first to the 100th epoch. The blue curve represents the training accuracy, while the orange curve represents the testing accuracy.

**Figure 13:**
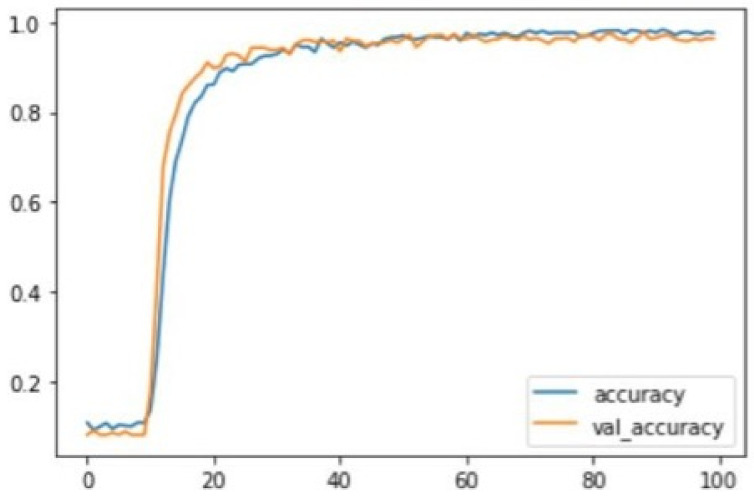
Graph of CNN model training and testing accuracy against the number of epochs.

From the graph Figure 13, it can be seen that the accuracy for both the cases starts at a very low percentage at the beginning of the testing and then rapidly increases around the tenth epoch at a rate of about 30%, after which it stabilizes and maintains a range of almost 100% as it approaches the end of training and testing. The loss of both the training and testing is illustrated in the figure below. The loss is plotted on the *Y*-axis, while the epochs are plotted on the *X*-axis. The blue curve represents the loss in training, while the orange curve represents that in testing.

**Figure 14:**
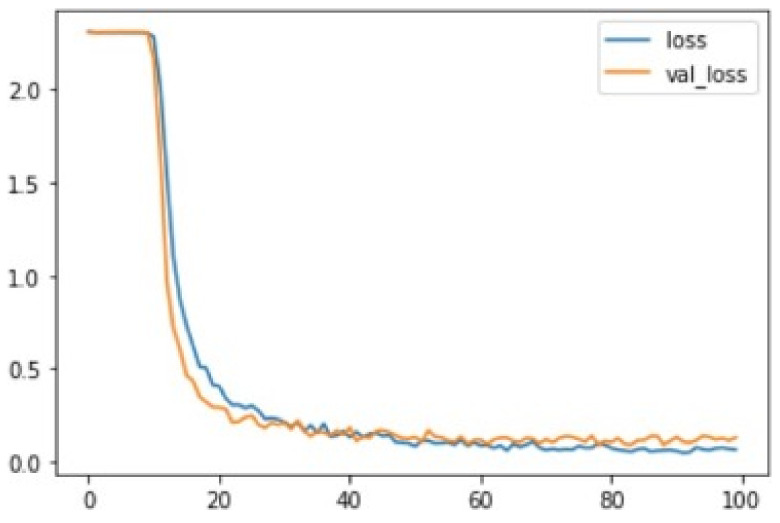
Graph of loss against the number of epochs in training and testing of the CNN model.

From this graph Figure 14, it can be seen that the loss in both cases is 2.0. The loss then decreases at a high rate from about the tenth to around the thirtieth epoch. The loss then begins to stabilize gradually, almost approaching zero toward the end of the training and testing.

Further analysis of these results was carried out using a confusion matrix for multi-class datasets, as shown below. In the confusion matrix, the rows represent the actual values in the dataset, while the columns represent the predicted values. The diagonal of the confusion matrix represents the value of the correct datasets which were rightly expected. In contrast, the values of the diagonals represent wrongly-predicted values in each class of dataset. To better show the patterns in the behaviour and performance of the model, the highest value at each level of class in every row/column is shaded with a darker colour.

**Figure 15:**
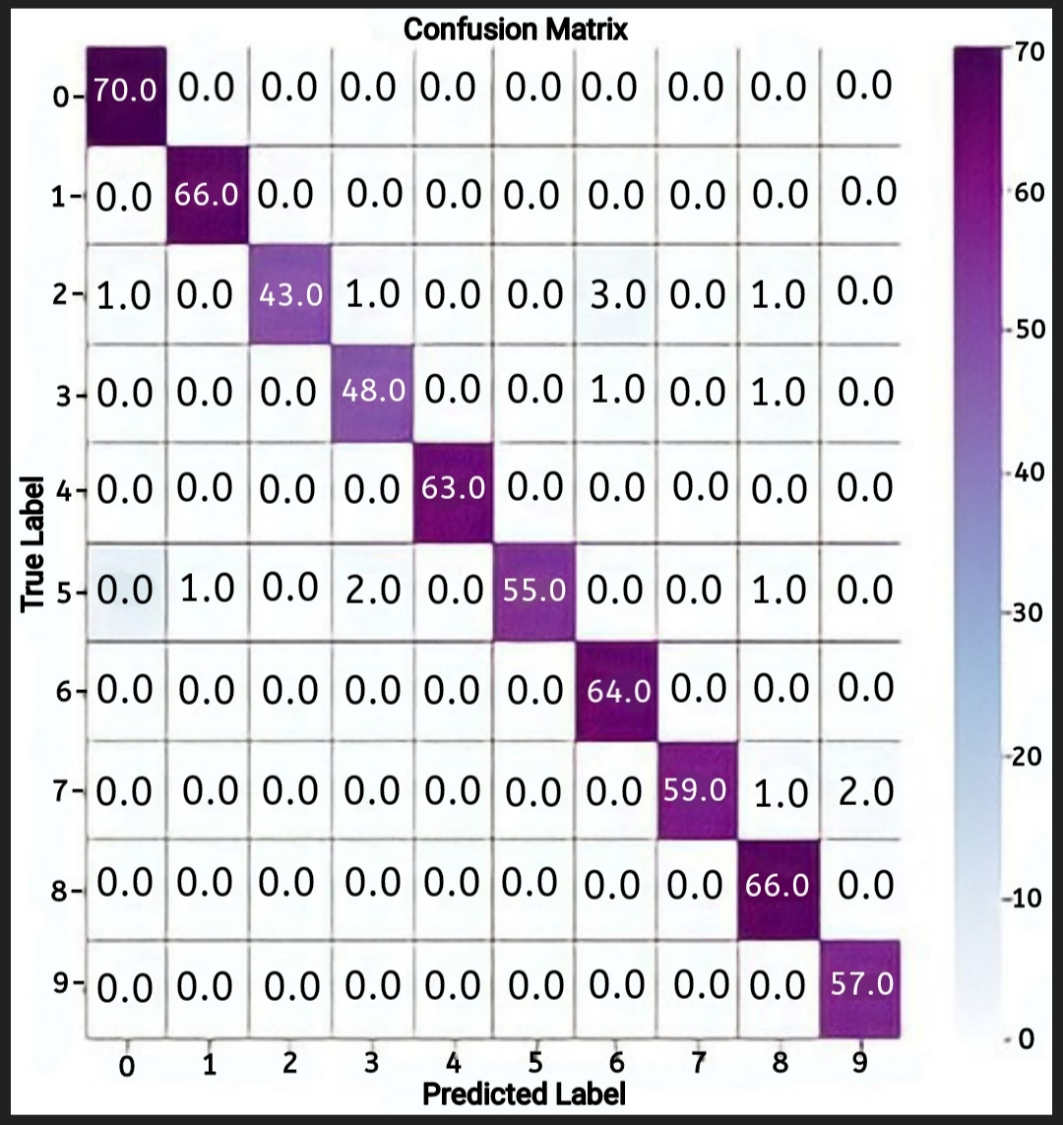
Confusion matrix for the CNN model.

In Figure 15, the CNN model’s confusion matrix is given above to show the performances on the classification task.

From the multi-class confusion matrix shown in the figure, the highest values for each class level lie on the diagonal. This indicates that most of the correct values in the dataset were predicted correctly by the CNN. On the other hand, most diagonal values are zeros, while others are significantly low values. This shows that there were very few errors in the predictions made by the developed CNN model in most instances. The F1-score had an accuracy of 96%. The average macro-level average precision and recall for the model were 97% and 96%, respectively. The average weighted precision and recall were 97% and 96%, respectively. Thu, we can say that the convolutional neural network model developed in this experiment showed consistent training and testing accuracy and attained a high level of reliability in its predictions. Therefore, it can be concluded that the performance of the convolutional neural network was exceptionally good for the prediction of numerical sign language using pre-processed data.

#### 4.1.3. Building and Training of the LSTM Deep Learning Model Using the Pre-Processed Dataset

The same pre-processed input dataset that involved importing NumPy arrays from Kaggle.com mapped and classified as described in the above procedure was applied again to develop, train, and test the LSTM model. Using an Adam optimizer learning rate of 0.001, the results were compiled and fitted into the LSTM model. The training and testing of the model were carried out over the course of 100 iterations.

**Figure 16:**
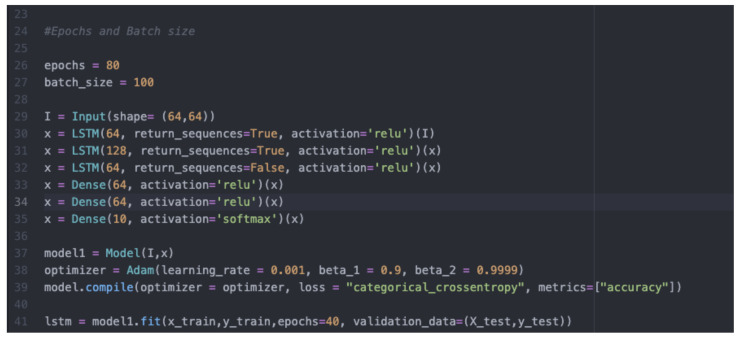
Building the LSTM model.

Some configuration parameters for LSTM are given in Figure 16.

#### 4.1.4. Evaluation of the LSTM Model

As in the case of the CNN model, the training accuracy of the LSTM model involves more than testing the accuracy. The difference between testing and training accuracy is not likely to fall below 5% under normal conditions. Both the training and testing accuracy generally vary throughout the process; this variation can either be positive or negative at different stages of the training or testing process based on the performance behaviour of the developed model. In this experiment, the training and testing of the LSTM model were both left to run and were recorded for all 40 iterations. During the training and testing process the accuracy was 35% and 32% respectively. As the training progressed, the training accuracy continued to increase following a very irregular pattern; the rate of increase was not stable. In addition, while the testing accuracy continued to increase as testing approached the fortieth iteration, the rate of increase was very irregular. The lowest training accuracy for the LSTM model was 29.0%, while the highest training accuracy was 49.4%, for a range of 20.5%.

On the other hand, the lowest testing accuracy for the LSTM model was 25.5%, and the highest testing accuracy was 48.47%, for a range of 22.97%. The loss in both training and testing for the LSTM model had a relatively decreasing tread with the iteration. Again, the decrease was in a very irregular manner. The figure below shows a graphical illustration of the behaviour of the LSTM model in both training and testing accuracy. The accuracy is plotted on the *Y*-axis in the graph, ranging from 0–100% (where 1.0 represents 100%), while the number of epochs is plotted on the *X*-axis from the first to the fortieth iteration. The blue curve in the graph represents training accuracy, while the orange curve represents testing accuracy.

**Figure 17:**
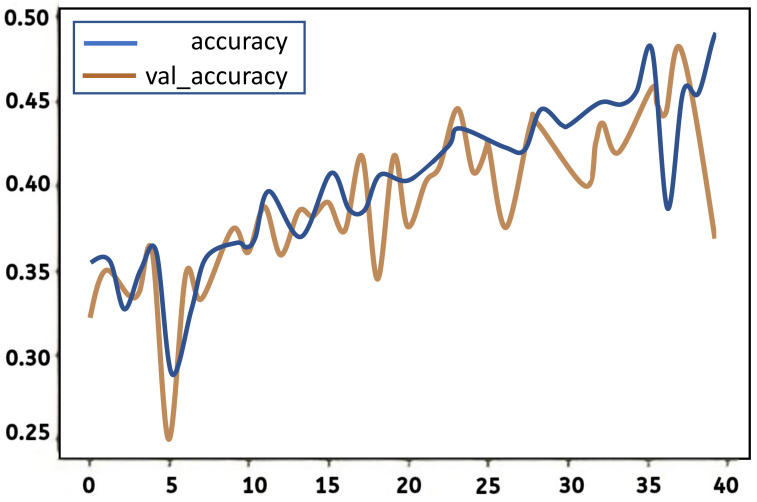
Graph of LSTM model training and testing accuracy against the number of iterations.

A graph shows trainign and testing accuracy rates against the number of iterations for the LSTM model in Figure 17.

The graph shows that although both the training and testing accuracy increase as the number of epochs increases, the curves have a very irregular pattern. According to the graph, the lowest training and testing accuracies occurred at around the fifth iteration, while the highest precision for both training and testing lies at around the 36th and 40th iteration, respectively. Neither curve seems to show stable perforce at any range of iteration testing. The loss in both the training and testing is illustrated in the figure below. The loss is plotted on the *Y*-axis of the graph, while the epoch is plotted on the *X*-axis. The blue curve represents the loss in training, while the orange curve represents the loss in testing.

**Figure 18:**
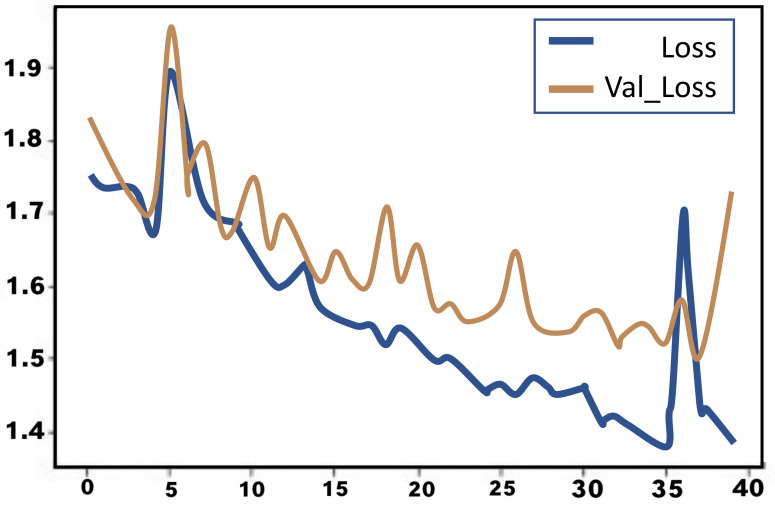
Graph of loss against the number of epochs in training and testing of the LSTM model.

A graph shows trainign and testing loss function against the number of epochs for the LSTM model in Figure 18.

Although this graph shows a relatively decreasing trend in the loss during both training and testing, the trend is very irregular, with random steep increases and decreases all along the curve. This brings a sense of unpredictability to the dataset.

A deeper analysis of these results was carried out using a confusion matrix for multi-class datasets, as shown below. In the confusion matrix, the rows represent the actual values in the dataset, while the columns represent the predicted values. The diagonal of the confusion matrix represents the values of the correct datasets which were rightly expected. In contrast, the weights of the diagonals represent the wrongly predicted values in each class of datasets. To better show the pattern in the behaviour and performance of the model, the highest value at each level of class in every row/column is shaded a darker colour.

**Figure 19:**
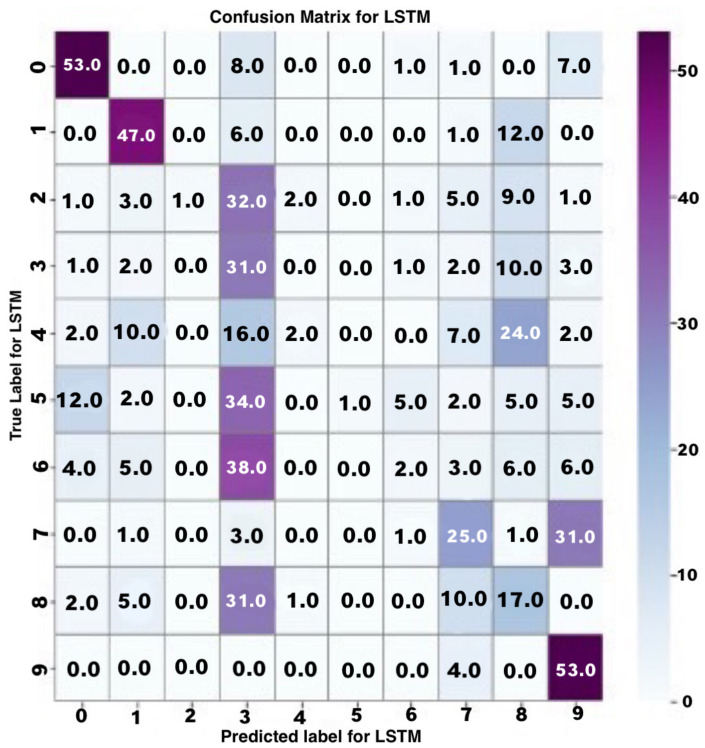
Confusion matrix for the LSTM Model.

In Figure 19, the LSTM model’s confusion matrix is provided to show the classification performances.

From the confusion matrix, it can be seen that most of the highest predicted values in the different levels of the classes are randomly distributed throughout the matrix. There is no noticeable pattern of values at the diagonal of the matrix, which shows that there is no reliable or stable pattern in the correctness of the LSTM model in predicting numerical sign language. The macro-level average precision and recall of the model were 52% and 38%, respectively. The average weighted precision and recall were 52% and 37%, respectively. The performance of the LSTM model in both training and testing was generally poor. Comparing the predictive performance of the LSTM and CNN models using pre-processed numerical sign language data, the CNN model performed exceptionally well compared to the LSTM model.

### 4.2. Facial Expressions Combined with Pose Signs

This section covers the results for the deep learning models when used to detect simple common message poses such as those for Hello, Good, Thanks, Sorry, and Happy. Instead of using pre-processed data, in this case the data collected under this part relied on collection of different facial and pose landmarks using a computer webcam. This involved installing and exporting the required dependencies in python and then using a MediaPipe holistic to extract key points from the different facial and pose landmarks. Then, the way in which fundamental point values for the different landmarks would be carried out at different points in the model was determined. The dimensions for these images were 150, 30, 1662. This means that the length was 150 while the width was 30, which is non- uniform. As mentioned earlier, the CNN model only accepts images with equal dimensions for height and width. Therefore, the images dataset collected for this model was only used to develop, train, and test the LSTM model. It should be noted that the data set was a 70/30 Spector, with 70% of the data used for training and 30% for testing. The dataset was further processed and reshaped into LSTM-compatible data and scaled to convert all the bales into intervals of (0,1). Using an Adam optimizer learning rate of 0.01, the results were compiled and fitted into the LSTM model. The training and testing of the model were carried out over the course of 2000 iterations.

#### Evaluation of the Second LSTM Model

During the training and testing of the LSTM model, the first training accuracy was 12.2%, while the first testing accuracy was 11.22%. The training accuracy changed rapidly, with an increase of about 10% during the first few iterations. A training accuracy range between 21–29% was then maintained for the rest of the epoch with minimal variance. An exciting pattern occurred with the testing accuracy throughout the testing, with the accuracy varying for the first few epochs, attaining an accuracy of 22% at the fourteenth epoch, and maintaining the same value up to the 34th. After that point, the accuracy dropped further to 11.11% and remained constant for the rest of the epochs. The figure below shows a graphical representation of both training and testing accuracy against the number of epochs. The accuracy is plotted on the *Y*-axis, while the number of epochs is plotted on the *X*-axis. The blue curve in the graph represents training accuracy, while the orange curve represents testing accuracy.

**Figure 20:**
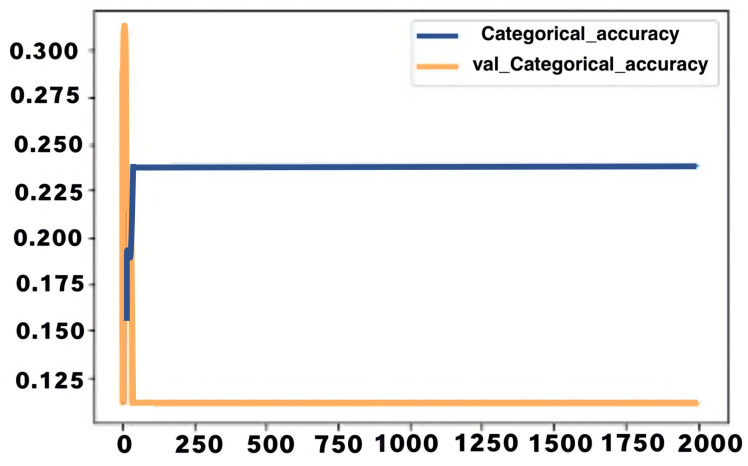
The second LSTM training and testing accuracy against the number of epochs.

In Figure 20, training an testing accuracy rates (*y*-axis) are given in the diagram against the epochs number (*x*-axis).

The graph shows that the LSTM model maintained an average training accuracy of about 23%, with almost zero variance for a significant part of the training procedure. The system maintained a very low testing accuracy of 11% for a significant portion of the testing procedure. The loss in both cases has related behaviour, starting at a very high value and then dropping suddenly. The initial loss values for training and testing were 316 and 10,449, respectively, while the final value in both cases was 1.6. The figure below shows a graphical representation of the loss in training and testing. The loss value is plotted on the *Y*-axis, while the number of epochs is plotted on the *X*-axis. The blue curve represents training loss, while the orange curve represents testing accuracy.

**Figure 21:**
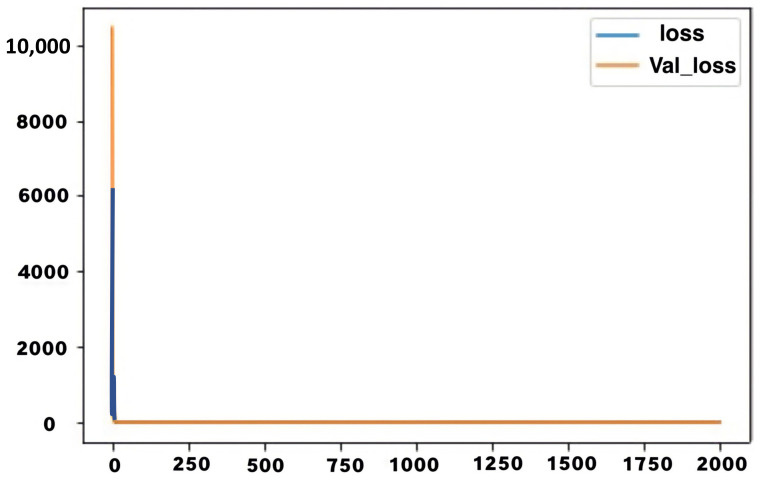
Loss against the number of epochs in training and testing of the second LSTM model.

In Figure 21, the accuracy loss function is represented against the number of epochs in training and testing for the second LSTM model.

**Figure 22:**
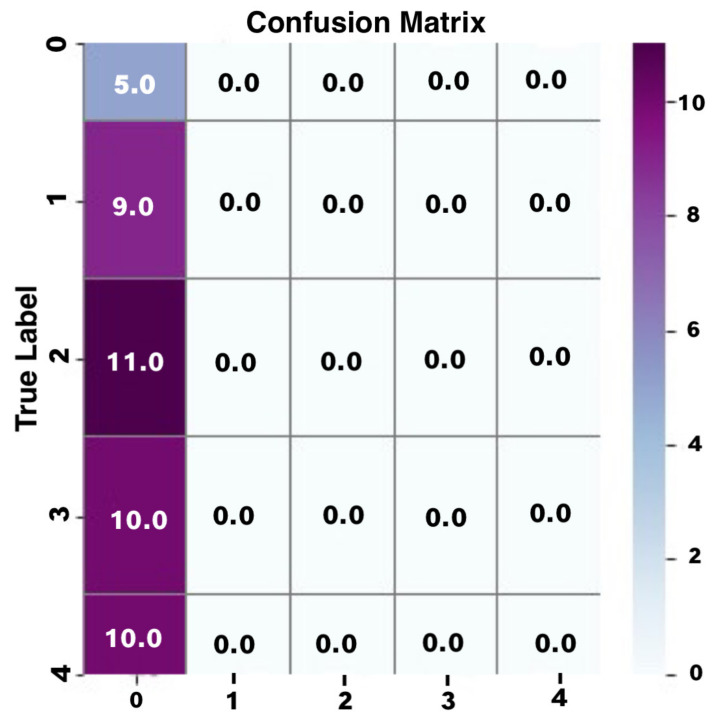
Confusion matrix for the second LSTM model.

A confusion matrix analysis was used to further analyse the behaviour of the LSTM model when used to predict simple common sign language messages. Figure 22 shows the results of the confusion matrix analysis as below.

From the confusion matrix diagram above it can be seen that many values were wrongly predicted, with the diagonal values all showing zeros; this indicates that no signs were predicted correctly. The macro-level average precision and recall for the model were 2% and 20%, respectively. The average weighted precision and recall were 1% and 11%, respectively. This indicates that the second LSTM model generally showed poor performance in training and predicting the simple common sign language signs.

## 5. Discussion

In this section, the results obtained in the course of this research regarding the objectives, hypotheses, and relevant secondary topics are discussed in depth, along with the role of the above results and the provided literature analysis in answering the research questions at issue. To this end, this section is divided into three main parts, namely, the interpretation of the results, the implications of the results, and the limitations of this study. The interpretation section elaborates on what the obtained results mean; the implications section discusses the relevance of the research and its relationship to existing findings; finally, the limitations section covers those aspects of the study where possible constraints may have hindered the investigation from reaching a complete evaluation or definite conclusions.

### 5.1. Interpretations of the Results

This research aimed to answer the main research question of how effective deep learning and computer vision models could be in narrowing the gap between people with speech impairments who can only use sign language and the general public who cannot speak using sign language. The present study aimed to determine which deep learning model among LSTM and CNN was the most efficient in interpreting or predicting British Sign Language. The process of answering the above questions involved a critical analysis of the existing literature concerning deep learning as well as a coding experiment. The coding experiment involved collecting different datasets, using those datasets to develop two deep learning models (CNN and LSTM), training them, and testing their efficiency in predicting British Sign Language. Below is a summary of the findings from the coding experiment.

#### 5.1.1. Summary of the Results

The coding experiment was divided into two main approaches depending on the dataset used. The first approach involved developing deep learning models to predict numerical sign language gestures using pre-processed data from Kaggle.com. The second approach involved developing deep learning models to predict simple standard messages (facial expressions combined with pose signs) using a dataset obtained using a computer webcam. In the first approach, both CNN and LSTM models were successfully developed, trained, and tested, and their performance was evaluated using a multi-class confusion matrix. Based on this approach, the testing and training accuracy of the convolutional neural network rose to 98.8% and 97.4%, respectively. The training and testing accuracy increased as the number of iterations increased, showing a positive correlation. Based on the multi-class confusion matrix, the convolutional neural network model developed in this experiment showed consistent training and testing accuracy and attained a high level of reliability in its predictions thanks to a consistently high rate of correct numerical hand gestures predicted correctly by the model. The CNN model achieved average weighted precision and recall of 97% and 96%, respectively.

On the other hand, the training and testing accuracy of the LSTM model reached a maximum of 49.4% and 48.7%, respectively. Although accuracy increased with the number of iterations, the curves had a very irregular pattern. Based on the multi-class confusion matrix, the LSTM model developed in this experiment showed inconsistent training and testing accuracy and attained a low level of reliability in its predictions. This was because the model followed a very random prediction method, and as a consequence the correct prediction of signs was random. Furthermore, the number of correct numerical hand gestures which were predicted correctly was very low. The macro-level average precision and recall for the model were 52% and 38%, respectively, and the respective average weighted precision and recall were 52% and 37%. From this first approach, it can be concluded that the convolutional neural network performed significantly better than the LSTM. This better performance was present in all aspects, including testing and training accuracy, precision, and reliability.

Regarding the second approach, only the LSTM model was successfully developed, trained, tested, and evaluated for performance using the multi-class confusion matrix, as the CNN model could only accept 4D input data and images with uniform length and width dimensions. These four dimensions include an image’s batch size, length, width, and depth [47]. The dataset obtained from the webcam mainly had key points for the face, hands, and pose, as opposed to images; thus, it was not possible for the CNN to extract the necessary four dimensions. The performance of the LSTM model saw the training and testing accuracy increase by an average of 23% and 11%, respectively. The exciting aspect of this experiment is that the training accuracy increased significantly within the first few iterations, then maintained an average of 23% throughout the remaining iterations.

On the other hand, the testing accuracy increased within the first few iterations and then decreased suddenly, maintaining an average accuracy of 11%. Based on the multi-class confusion matrix, this LSTM model showed inferior prediction ability and unreliability in the prediction of sign language, as demonstrated by the lack correctly predicted signs. All the values of the diagonal of the matrix were 00, indicating that no signs in the simple standard message (facial expressions combined with pose signs) were correctly predicted. The macro-level average precision and recall for the model were 2% and 20%, respectively, while the average weighted precision and recall were 1% and 11 %, respectively. This indicates that the second LSTM model generally showed poor performance in both training and prediction of simple common sign language signs.

#### 5.1.2. Interpretation of the Results

The obtained these results clearly show that the convolutional neural network performed better than the LSTM model, indicating that the CNN model is the best model among the two in predicting British Sign Language. The CNN model’s 98% training accuracy, 97% testing accuracy, and 97% precision with only 100 iterations show that this deep learning model has high potential to predict British Sign Language efficiently. These results confirm the first hypothesis, namely, that deep learning and computer vision models can effectively improve the communication ability of speech-impaired people, offering high accuracy, precision, and reliability. In addition, the results support the second hypothesis of this study, as the CNN model showed better accuracy, precision, and reliability compared to the LSTM model.

The training and testing accuracy of the CNN model increased with an increased number of training iterations, thus establishing a positive correlation. Increasing the number of iterations increased the training duration and provided the model with more time to learn from the data and make correct predictions on that basis. From the obtained results, we can confirm that the accuracy of the CNN model increases with the increased duration of the training period as determined by the number of iterations and images. That the LSTM performed poorly with the same data types and under the same setting as the convolutional neural network, indicating that different deep learning models perform differently with different tasks and coding.

#### 5.1.3. Interrelation of the Findings and Literature Review

In answering whether deep learning models and computer vision models can help narrow the gap between the speech-impaired community and the general public, the results of these experiments show that certain deep learning models, such as CNN, perform well. Achieving an accuracy of 98% within only 100 iterations is a good indicator that the CNN deep learning model can help narrow this gap if further modifications can be added using more iterations. These findings confirm what other researchers in the literature review had argued in their studies. This includes acknowledging that a gap needs to be eliminated between speech-impaired community members and the rest of the community, as pointed out by [24,47]. The success of developing a deep learning model that could predict sign language at such a high level of accuracy, precision, and reliability confirms the findings of other researchers [27] that the deep learning model can be relied upon to narrow this gap [27]. Although the actual experiment conducted in this research did not compare efficiency between conduct-based human action recognition and vision-based human action recognition, this research agrees with their argument that the latter could be preferable. This is because (as [17] stated in his study) the deep learning model can be a very reliable, relatively inexpensive, and efficient approach that best fits sign language recognition, as further confirmed in this study by the CNN model.

It should be noted that developing this model was economical, and required the purchase of no additional hardware components. Although the university had provided the necessary resources for all the experiments, the cost of the required resources would have been only GBP 60 for a graphics card-enabled laptop). Few specialised professional skills were necessary, unlike the electronic and mechatronic engineering required to use conduct-based models (for instance, the model developed by Abraham & V, 2018 required installation and modification of sensors and a mechanism that links flex angles, voltage, and resistance). In addition, developing the model did not take very much time compared to the demands of building a conduct-based model. These practical differences confirm that the deep learning model can effectively narrow the gap between the speech-impaired community and the general public by assuring high accuracy, precision, and reliability at low cost. Considering the relationship between the performance of the CNN model compared to the LSTM, the findings of this research agree with the various authors that have pointed out that CNN performes well on sign language prediction tasks. In [27], a human action recognition model for Indian sign language was developed and attained 99% accuracy. A different CNN model [32] was developed for Italian sign language detection, achieving an accuracy of 91.7% and a score of 0.789 on the Jaccard index during the Chalearn spotting competition in 2014, which involves the detection of people’s gestures. The present research agrees with their recommendation that CNN models are reliable for automatic sign language recognition. The findings in the present research further agree that CNN testing and training accuracy increases with an increased number of iterations and with an increase number of images in the datasets, improving the training duration [4]. The models developed in their research attained 99.94 and 97.62 training and testing accuracy, respectively, which is very close to the findings in this paper. These results are close to what [47] achieved in their models as well, namely, a 99.8 and 97.8 accuracy in predicting numerical and alphabetical Indian sign language.

The poor performance of the LSTM model can be explained by reference to limitations previously pointed out by other authors. For instance, [48] pointed out that it can be challenging to train the LSTM model, as memory-bandwidth-bound computation is required. This imposes hardware disadvantages and limitations on the applicability of LSTM in image recognition and prediction. In [49], the authors state that for efficient application of the LSTM model in image classification and recognition, additional actions such as extraction of image features may be necessary before the model is applied. Several other studies have pointed out that the LSTM model functions better in human action recognition when integrated with other boosting models or in composition with different parameters or models. This model consisted of a convolutional neural network integrated with an attention mechanism to retrieve spatial features and bio-inspired deep learning with long short-term memory. This model showed good performance and required a lower processing time. Therefore, it can be concluded that the LSTM model is able to perform better if additional parameters and modifications can be incorporated to curb its limitations.

### 5.2. Implications of the Results

The findings of this research add to the evidence demonstrated in previous research that deep learning and computer vision-based models can help narrow the gap between the speech impaired community and the general public. This can be seen in the success of building deep learning models. The convolutional neural network model developed here was able to predict British Sign Language hand gestures with high accuracy, precision, and reliability for numerical sign language. The development of this model was economical, time-efficient, and required a relatively basic skill level compared to conduct-based models. The accuracy of the model was found to have a positive correlation with the number of input images. This proves the potential to build a more advanced deep learning convolutional neural network model able to predict British Sign Language with high accuracy and prediction. The success of such a model would enable speech-impaired people to express themselves using sign language while the model translates their message into written text that anyone can read and reply to in text.

The results of this research further add to the existing research showing that convolutional neural networks perform well in the prediction of sign language compared to LSTM models. The CNN model showed attainment of high accuracy in training and testing along with high precision, consistency, and reliability. This matches the results published by other researchers in their attempts to use the CNN model in the prediction of different sign languages. Therefore, the implication is that the UK government or private actors could choose to implement a deep learning model that applies CNN for recognition and prediction of British Sign Language to help ease communication between people with speech impairments and the rest of the community. In addition, any research intending to develop a machine learning model for recognition and prediction of any other sign language in the world might begin by trying the CNN model, as it has proven to be successful in many different settings. Other scholars intending to perform research on deep learning can apply this research in various ways as well.

The findings of the present study and its research implications help to improve knowledge through its potential applications in new research and in the advancement and confirmation of the validity of already-existing research. It should be noted that the success of this research represents the fulfillment of future research recommendations outlined by previous authors. For instance, [27] suggested that future research could focus on comparing the performance of different deep learning models in human actions and sign language recognition and prediction. Other researchers, such as [27,32], called for the need for research on the use of CNN to predict other sign languages and improve the existing findings. In this regard, the present research compared the performance of CNN and LSTM models in terms of their accuracy, consistency, and precision, and further involved the application of the developed model in the recognition and prediction of British sign language.

The practical application of these research findings lie in developing a CNN sign language recognition and prediction system to be used in public service offices and business organizations such as hospitals, banks, wholesale shops, and schools. Such a convolutional neuron network could enable speech-impaired people to express themselves using sign language. The model would translate the message to a text form that the service providers can understand. In addition, the findings of this research could be applied to develop a universal CNN model that could recognize and predict different types of sign language from different locations all over the world. This would make it possible for speech-impaired people who use different sign languages to communicate. For example, a person using British sign language could speak with a person using Indian sign language, provided they can both read and understand text written in the same language.

### 5.3. Acknowledgement of Limitations

To achieve the objectives of this research two approaches were considered, which were based on the types of data required. The first approach, which involved developing both CNN and LSTM models for recognizing and predicting hand gestures in British numerical sign language, was successfully completed. This approach depended on pre-processed data obtained from the Kaggle.com website. However, the second approach, which required the development of both CNN and LSTM models for predicting simple common messages (facial expressions combined with pose signs) was not fully accomplished, as only the LSTM model was developed. The main limitation preventing the full achievement of this approach was the lack of CNN compatibility. Unlike the first approach, which involved using pre-processed data from Kaggle.com, this approach depended on a dataset retrieved using a computer webcam. Using the webcam involved extracting specific key points from the face, hands, and pose landmarks, and the CNN model could not accept data in this form as it applied four-dimensional data consisting of the image’s batch size, length, width, and depth. The data obtained by the webcam consisted of three-dimensional data informed of key points of the different landmarks, and not images; thus, the CNN could not extract the fourth dimension, that is, the depth of vision. This aspect of CNN limited development of a CNN model to predict simple common messages (facial expressions combined with pose signs) and compare its performance with that of the LSTM model. If this had not been the case, and the CNN model had accepted the data, it would be possible to make a more informed conclusion as to the better performance of the CNN in predicting hand gestures and simple standard messages (facial expressions combined with pose signs). However, considering the results with respect to the performance of the CNN model in both this experiment and other studies referred to in the literature review, it is justified to assume that the CNN model would have performed well in the prediction of simple common messages (facial expressions combined with pose signs).

## 6. Conclusions

### 6.1. Summary and Findings

This paper aimed to develop an efficient deep learning model for the detection, understanding, and translation of British sign language to written text. The research was based on background information pointing out that this problem represents a significant communication gap between the speech-impaired (that is, deaf and mute people) and the general public. These two groups of people use a different form of language to communicate; speech-impaired people use sign language, while other people generally use spoken language. This background information points out that it is difficult for most people to learn sign language, and speech- impaired people are not able to learn spoken languages. It should be further noted that sign language falls into different types depending on geographical locations and ethnic divisions. This means that it is not possible for all speech-impaired people to communicate using common sign languages. This gap could quickly be narrowed if these groups could both be linked using written text messages. Although other methods can be applied to link communication under these circumstances, such as using a human translator, conductor-based machines for sign language, and deep learning/computer vision models, this research points out that the latter is the most effective.

The primary approach of this research was to develop two deep learning models, a long short-term memory (LSTM) model and a convolutional neural network (CNN) model, and compare their performance. The experiment involved collecting the required datasets, using them to develop the models, training and testing the two models, and applying a multi-class confusion matrix to evaluate their performance. The parameters used for the comparison included training and testing accuracy and the systems’ respective precision and reliability/consistency in predicting sign language. The approach was then divided into two categories; the first used pre-processed data to predict hand gestures for British numerical sign language, and the second used a key points dataset to indicate simple common messages (facial expressions combined with pose signs). In the first approach, both the CNN and LSTM models were developed. The CNN model showed the best performance in all aspects, including accuracy, precision, and reliability, as stated in the research hypothesis. Furthermore, this model showed a positive correlation between training/testing accuracy and the length of the training period as determined by the number of iterations and images per dataset. This resulted in the CNN model attaining high accuracy. A more significant number of signs and more iterations could be applied to increase the training and testing accuracy; the model would then be applicable for accommodating more than one type of sign language, making it more efficient.

On the other hand, the LSTM model showed very poor performance in both categories of the experimental approach. This model attained very low accuracy, precision, and consistency in predicting the correct sign based on the multi-class confusion matrix. The most reasonable explanation for the poor performance of the LSTM was due to certain limitations that were pointed out in the literature review. For instance, this model can be difficult to train, as it requires a memory-bandwidth-bound computation which has hardware limitations. LSTM models depend on more complex frameworks to achieve good performance compared to CNN model. This research found that while LSTM models are better in classification of text data, for image data sets more input parameters may be needed. The performance of the LSTM model could be improved by integrating it with other models to curb its limitations. It was not possible for a CNN model to be developed in the second approach, as the dataset used was incompatible with the requirements of a CNN. However, based on the results of the first approach as well as on the literature review, it can be assumed that the CNN model would have performed well in this second approach.

Therefore, this research concludes that convolutional neural networks perform better in recognizing and predicting British sign language than LSTM models. In addition, this research further concludes that the CNN model could be used to accommodate more than one set/type of sign language recognition prediction. The findings of this research answer the question of which deep learning models perform better in attempting to narrow the gap between speech-impaired people and the general public.

### 6.2. Contribution to This Research

The success of this research is beneficial to people from the speech-impaired community, the government and economy of the UK, and to scholars and researchers worldwide. The proposed CNN sign language recognition and prediction model would help people from the speech-impaired community to interact more easily with the general public, promoting better communication in services provided in public offices and organizations that are not conversant with sign language. It would be possible for people with speech impairments to feel as appreciated and able to participate in the community as anyone else. The government could implement such a model in its offices and operations in order to take care of people with disabilities. This would enable speech-impaired people to easily express their needs to the government, promoting political harmony. Speech-impaired people would be able to join more workplaces and work more efficiently, earning more income and contributing to economic growth. For scholars and researchers, the findings of this research can serve as the basis for future works.

### 6.3. Future Recommendations of This Study

This research points to the following recommendations. A live-trained CNN model can be developed based on insights from this research. This is because the CNN model has proven to perform better than LSTM based on this study. Such a model should convert, detect, interpret, or predict sign language from video and display it through a special screen. It should be able to pre-process data automatically. Sign language recognition and prediction should be effectively instantaneous. The model should be fitted with high-definition digital cameras to capture high-quality videos that the CNN model can process.

Developers and sponsors can adopt the finding of this research on convolutional neural networks and modify it for the benefit of society at large. This could include building a more comprehensive model that accommodates the entire set of British Sign Language and using it to help speech-impaired people to more easily communicate in public offices or facilities. A further recommendation is that future research develop a CNN model that accommodates different sign languages and compare its effectiveness. Future research could additionally try to improve the performance of LSTM by combining it with other models and then comparing the performance of the LSTM composite models with CNNs.
